# Sensory neuron–associated macrophages as novel modulators of neuropathic pain

**DOI:** 10.1097/PR9.0000000000000873

**Published:** 2021-03-09

**Authors:** Conceição Elidianne Aníbal Silva, Rafaela Mano Guimarães, Thiago Mattar Cunha

**Affiliations:** a Center for Research in Inflammatory Diseases (CRID), University of São Paulo, São Paulo, Brazil,; b Graduate Program in Basic and Applied Immunology, Ribeirão Preto Medical School, University of São Paulo, Ribeirão Preto, São Paulo, Brazil,; c Department of Pharmacology Ribeirao Preto Medical School, University of Sao Paulo Ribeirão Preto, São Paulo, Brazil. University of Sao Paulo, São Paulo, Brazil

**Keywords:** Neuropathic pain, Primary sensory neurons, Macrophages, Cytokines, Chemokines

## Abstract

The peripheral nervous system comprises an infinity of neural networks that act in the communication between the central nervous system and the most diverse tissues of the body. Along with the extension of the primary sensory neurons (axons and cell bodies), a population of resident macrophages has been described. These newly called sensory neuron–associated macrophages (sNAMs) seem to play an essential role in physiological and pathophysiological processes, including infection, autoimmunity, nerve degeneration/regeneration, and chronic neuropathic pain. After different types of peripheral nerve injury, there is an increase in the number and activation of sNAMs in the sciatic nerve and sensory ganglia. The activation of sNAMs and their participation in neuropathic pain development depends on the stimulation of pattern recognition receptors such as Toll-like receptors and Nod-like receptors, chemokines/cytokines, and microRNAs. On activation, sNAMs trigger the production of critical inflammatory mediators such as proinflammatory cytokines (eg, TNF and IL-1β) and reactive oxygen species that can act in the amplification of primary sensory neurons sensitization. On the other hand, there is evidence that sNAMs can produce antinociceptive mediators (eg, IL-10) that counteract neuropathic pain development. This review will present the cellular and molecular mechanisms behind the participation of sNAMs in peripheral nerve injury–induced neuropathic pain development. Understanding how sNAMs are activated and responding to nerve injury can help set novel targets for the control of neuropathic pain.

## Key Points:

1. Sensory neuron–associated macrophages (sNAMs) are involved in the pathophysiology of neuropathic pain through the production of proinflammatory and pronociceptive mediators.
2. Sensory neuron–associated macrophages are activated in the sensory ganglia after peripheral nerve injury mainly by PRRs and chemokines.
3. Sensory neuron–associated macrophages also produce anti-inflammatory mediators that counteract neuropathic pain development such as IL-10.
4. Understanding the interactions between injured sensory neurons and sNAMs can provide novel targets for neuropathic pain control.

## 1. Introduction

According to the current IASP definition, pain can be defined as “an unpleasant sensory and emotional experience associated with, or resembling that associated with, actual or potential tissue damage.”^202^ In other words, pain is a personal complex experience that includes the conscious perception of a stimulus capable of generating tissue damage and may further depend on cognitive and emotional components.^37,91,216,224^ Whereas acute pain has a protective function to avoid potential damage, chronic pain can be maladaptive and pathological, and it represents one of the most prevalent and disabling health conditions in modern society.^136,202^ Neuropathic pain is a type of chronic pain characterized by injury or disease that directly affects the somatosensory nervous system, including peripheral fibers and central neurons.^202^ Epidemiological studies estimate that this pathology affects an average of 7% to 10% of the general population, being one of the most prevalent health problem.^38,110,190^ The mechanisms involved in the development and maintenance of neuropathic pain were initially characterized as a neuronal dysfunction. Indeed, after nerve injury, a series of modifications occur across the pain pathway that includes alterations in ion channel expression and function, upregulation of neurotransmitters, and their receptors, which leads to a state of neuronal hyperexcitability.^13,43,46,50,54,61,77,119,145,176,205^

Currently, there is a growing body of evidence indicating that the cause of neuropathic pain is not restricted to changes in neuronal activity but may involve a network of interaction among neurons, glial, and immune cells.^37,48,78,86–88,240^ These cells may interact with neuronal cell bodies and their fibers distributed throughout the peripheral and central nervous system (CNS) in both pathological or homeostatic conditions. In the case of neuropathic pain, when nerve integrity is affected, immune/glial cells, which may be resident or recruited to the injured tissue, and distally to the sensory ganglia and spinal cord, are activated and release inflammatory mediators that strongly modify neuronal function, culminating in alterations of painful perception.^165,223,236^ Among the immune/glial cells, macrophages emerge as one of the most important cell subpopulations involved in neuroimmune interactions associated with neuropathic pain.^57,161,225^ This review discusses the current evidence regarding the cellular and molecular interactions between primary sensory neurons and resident macrophages associated with these peripheral neurons, known as sensory neuron–associated macrophages (sNAMs), that might play a crucial role in the development of neuropathic pain. The role of infiltrating monocytes in the site of nerve injury is also discussed. Finally, we pointed out additional mechanisms by which peripheral macrophages may also counteract neuropathic pain development.

## 2. Neuron associated–macrophages: their origins and fate

Tissue-resident macrophage populations are present in a variety of organs across the body.^166,228,240^ Although some characteristics and functions are shared among different macrophage populations, such as homeostasis maintenance and tissue protection, these cells exhibit high functional plasticity and thus have several specialized functions in each different niche/tissue.^58–133,133–136,138–166^

Historically, distinct subpopulations of macrophages have been defined according to the anatomical location and surface markers; however, this definition has been recently expanded to subset-specific gene expression signatures^181^ and ontogenies of these cell populations.^56^ It was known that monocytes newly released from bone marrow colonize various tissues, and once mature, they may become resident macrophages with specific features. It is currently accepted that most cells in the hematopoietic compartment are regularly renewed from adult hematopoietic stem cells (HSCs); however, recent findings demonstrate that resident macrophages can self-maintain independently of HSCs because they may have an embryonic origin. In this scenario, it is known that, at least in mice, tissue macrophages are derived from 3 different developmental sources.^60,70,166,210^ Macrophages firstly appear in the yolk sac (YS) during initial fetal development without monocytic intermediates and then colonize various embryonic tissues.^179^ In the embryonic period 8.5 (E8.5), macrophage precursors from the YS and HSCs migrate to the fetal liver and give rise to the first monocyte cells in E12.5.^79,142^ After birth, HSC in the bone marrow produces Ly6C+ monocytes, which can migrate to different tissues and differentiate into macrophages.^76,220^

Based on this, some groups have performed extensive characterizations of resident macrophages in the most diverse tissues, based not only on the anatomical location and profile of phenotypic markers but also on the transcriptional and ontogeny profile. In this sense, Gomez Perdiguero et al.^63^ proposed that macrophages of the liver, lung, and epidermis are originated from YS-derived erythro-myeloid progenitors. The CNS also has resident macrophages with specific characteristics, including self-sustainability and proliferation. Besides microglia, meningeal, perivascular, and choroid plexus macrophages are considered CNS interface cells that appear to be derived from the YS, demonstrating that different populations of CNS macrophages share similar ontogeny.^59,62^

In addition to the macrophages residing in the CNS, peripheral nerves also contain resident macrophages.^113^ These macrophages are distributed in the large peripheral nervous system interaction network and comprise one of the most important populations of myeloid cells associated with peripheral nervous tissue. For instance, in the rat, sciatic nerve macrophages constitute 1% to 4% of the total cell population.^162^ Conceptually, the term NAMs defines the subset of resident tissue macrophages that are closely associated with peripheral nerves in the most diverse tissues^113^ and can be characterized by the type of tissue and nerve in which they reside, origin, and self-renewal characteristic. The identification of macrophages in peripheral nerves occurred many years ago. In a pioneering study by Arvidson^10^ when examining the sciatic nerve of animals after the systemic injection of horseradish peroxidase, an enzymatic tracer that is widely distributed in most tissues, he observed through electron microscopy, cells with similar ultrastructural characteristics macrophages and located close to the epineurial and endoneurial. Later, Gehrmann et al.^55^ were able to demonstrate the presence of macrophages in the sciatic nerve and the dorsal root ganglions (DRGs), where the cellular bodies of sensory neurons are located. They confirmed the presence of macrophages in the DRGs by evaluating the expression of classic cell markers, such as CR3 and MHC-II, by immunohistochemistry reaction. Despite these data, only recently, sNAMs broad characterization was performed. Importantly, it was found that sNAMs from different neuronal compartments (sciatic nerve, DRGs, and cutaneous intercostal fascial nerves) are mostly self-maintained in adult mice.^112,219^ Contrary, ontology analysis of sNAMs of the sciatic nerves revealed they are predominantly from late embryonic precursors that are slowly replaced by bone marrow–derived monocytes.^231^ Therefore, further studies are important to finally define the origin of distinct sNAMs from different neural niches. Transcriptome analysis also revealed that sNAMs share some characteristics with activated microglia. However, sNAMs-specific genes were also identified, including genes related to angiogenesis, collagen fibril organization, and peripheral nerve structural organization and axon guidance.^219^ This specific transcriptional profile of sNAMs is in line with their possible role in axon sprouting after peripheral nerve injury.^112^ Besides that, the participation of sNAMs in the pathophysiology of neuropathic pain has been extensively studied, and these studies will be discussed below.

## 3. The sensory neuron–associated macrophages in the development of neuropathic pain

Neuropathic pain, the focus of this review, can occur because of several stressors, such as viral infections, diabetic neuropathy, mechanical trauma, neurotoxic chemicals, spinal cord injury, stroke, and multiple sclerosis.^38,77,110,190,221^ Models of peripheral nerve injury are widely used to mimic neuropathic pain and most of the common clinical characteristics of this pathology. The development of neuropathic pain models has been fundamental for characterizing pathophysiological mechanisms and has shed new light on the preclinical evaluation of potential therapeutic interventions.^119^

The injury of primary afferent neurons conducts these cells to a hyperexcitability state. Thus, the action potentials generated at their endings are easily carried forward to the second-order neurons in the dorsal horn of the spinal cord and later to supraspinal brain regions, where the painful sensation is processed and interpreted.^18,43^ It is currently well accepted that the interactions of immune and glial cells within the peripheral nervous system and CNS regulate neuronal excitability and sensitize the pain pathway.^145,155^ When nerve integrity is disrupted, neuroimmune interactions occur early in the local of injury and register the initial trigger for neuropathic pain development. Resident cells, including Schwann cells and sNAMs, are responsible for the production of earlier inflammatory mediators that mediate the recruitment of immune cells to the injured nerve.^9,47^ After a peripheral nerve trauma, initial recruitment of neutrophils occurs, followed by the infiltration of inflammatory CCR2+ monocytes, which might be important to amplify the immune response.^1,93^ Studies show that systemic treatment with chemotherapy drugs, which are well known as neurotoxic, promoted an increase in the number of CX3CR1+ and CCR2+ macrophages/monocytes in the peripheral nerves.^146^ Several studies suggested that these locally activated macrophages are directly associated with a significant increase in the levels of inflammatory mediators, which sensitize primary afferent neurons and contribute to the development of neuropathic pain.^51,57,66,130,148,236^ For instance, Cx3cr1-deficient or Ccr2-deficient showed delayed development of mechanical hypersensitivity caused by the treatment with chemotherapy drug vincristine.^146^ Although resident and infiltrated macrophages/monocytes at the site of nerve injury are considered essential for the development of neuropathic pain, most of the studies that claimed this possibility lack specific tools targeting only these cells to confirm this hypothesis. Given these methodological limitations, many efforts have been made to develop specific tools to precisely manipulate peripheral (resident and infiltrating) vs central (eg, microglia)^44,45^.

One of those promising examples is a recently described mouse strain in which the suicidal gene Fas is under the control of the colony-stimulating factor 1 receptor (CSF1R) promoter, called macrophage-induced fas-apoptosis (MAFIA).^24^ In these mice, Fas ligand administration drives the death of CSF1R+ cells. Unlike CSFR1 selective antagonists, this drug fails to cross the blood–brain barrier, ensuring higher peripheral macrophage specificity. By taking advantage of MAFIA mice, Shepherd et al.^183^ showed alleviation of mechanical pain hypersensitivity caused by peripheral nerve injury. The authors implicated the reduction in infiltrated monocytes as responsible for the MAFIA mouse pain phenotype.^183^ On the other hand, more recently, it was shown that specific depletion of macrophages/monocytes at the site of nerve injury did not affect the development of neuropathic pain, excluding any participation of macrophages/monocytes in the local of nerve injury for the development of neuropathic pain.^235^ Thus, although the systemic depletion of peripheral macrophages/monocytes reduces neuropathic pain development,^31,161,193^ it is likely that these cells could be acting in tissues different from the local nerve injury.

Besides the peripheral nerves resident sNAMs, as we mentioned above, there are also resident sNAMs in the sensory ganglia (DRGs and TGs). The injury of peripheral nerves promotes several changes at the level of sensory ganglia, including a neuroinflammatory process characterized by activation/proliferation of glial cells (eg, satellite glial cells [SGCs]), and sNAMs. Early studies using different sciatic nerve trauma models described an increase in the number of macrophages/monocytes around the cell body of sensory neurons in the sensory ganglia in a time-dependent manner.^108,117,235,238^ Generally, the number of macrophages peaks form 5 to 10 days after sciatic nerve injury, retracting afterward.^117,128,200^ In chemotherapy-induced peripheral neuropathy, an accumulation of macrophages in the DRGs was also observed by some groups, whereas others did not observe any change.^89,104,133,139,141,238^ Although the reasons for this discrepancy are not immediately apparent, it could be related to differences in the doses of the chemotherapy drug used, schedules of treatment, and evaluated time points. There is another debate regarding whether the accumulation of macrophages in the sensory ganglia after peripheral nerve injury is due to the infiltration of blood monocytes or the local proliferation of sNAMs. Thus, further studies will also be necessary to clarify this point.

To dissect the participation of sNAMs in the sensory ganglia for the development of neuropathic pain, some strategies were applied. The intrathecal administration of minocycline reduced the number of sNAMs in the DRG after peripheral nerve injury, which was accompanied by the downregulation of inflammatory mediators reflecting on the reduction of mechanical pain hypersensitivity.^117^ A combination of genetic and pharmacological tools for conditional depletion of peripheral sNAMs/monocytes and microglia also prevented the development of pain hypersensitivity in a mouse model of spinal nerve transection.^165^ Targeting peripheral macrophage and microglia with CSFR1 inhibitor, a receptor involved in survival, proliferation, and differentiation of macrophages in different tissues, also reduced neuropathic pain caused by peripheral nerve injury.^121^ In addition, the clodronate-induced killing of sensory ganglia macrophages reduced neuropathic pain development caused by peripheral nerve injury (trauma) and chemotherapy (paclitaxel).^36,238^ Noteworthy, none of these treatments are selective for sNAMs in the sensory ganglia and also target infiltrating monocytes and/or microglia. Based on that, the same study that ruled out the contribution of nerve injury–infiltrating macrophages for the development of neuropathic pain provided evidence that sNAMs in the sensory ganglia play a critical role in this condition.^235^ However, we could not discard those peripheral monocytes could be acting in additional sites than the local of nerve injury. For instance, we recently found that after peripheral nerve injury, CCR2+ monocytes become adhered to the endothelial cells of the spinal cord microcirculation, and these cells could also have a role in central mechanisms of neuropathic pain^71^. Finally, it is important to mention that the discovery of specific cellular markers for sNAMs of the sensory ganglia that could differentiate them from other resident macrophages and monocytes would be essential to develop specific strategies to target only these cells and dissect their real contribution to neuropathic pain development.

## 4. Mechanisms of sensory neuron–associated macrophages activation and accumulation after nerve injury

As we mentioned above, after peripheral nerve injury, the activation/accumulation of sNAMs in the sensory ganglia (DRGs) seems to play an essential role in the development of neuropathic pain. Although it is not totally clear how the peripheral nerve injury leads to the distal activation/accumulation of sNAMs in the sensory ganglia, some possible mechanisms have been proposed.

### 4.1. sNAMs and innate immunity receptors

Like classical immune cells, macrophages can express different innate immunity receptors, such as Toll-like receptors (TLRs) and nucleotide-binding cytoplasmic oligomerization (NLRs) receptors.^26,41,108,160,195–197,233^ The large family of TLRs plays a critical role in immune responses by the recognition of pathogen-associated molecular patterns (PAMPs) and damage-associated molecular patterns (DAMPs),^2^ such as heat shock proteins, necrotic cells, and extracellular matrix components.^2,19,21,111,123,133,134,163,192,197,239^ Continuous activation or dysregulation of TLRs signaling may contribute to chronic disease states and have been involved in the pathogenesis of neuroinflammation, including in neuropathic pain development.^28,152,222^ In this sense, several studies have indicated that the activation/proliferation of microglia in the spinal cord, after peripheral nerve injury, that accounts for neuropathic pain development might depend on TLRs stimulation.^132^ For instance, Shi et al. demonstrated that spinal cord microglia activation after peripheral nerve injury depends on an unidentified endogenous ligand of TLR2 derived from damaged peripheral nerves.^184^ More recently, it was found that after peripheral nerve injury, GT1b ganglioside is axonally transported from the cell body of sensory neurons into the spinal cord and mediates neuropathic pain development through the activation of TLR2.^126^

Regarding the role of TLRs in sNAMs activation, TLR2 null mice also showed a reduction in the activation/accumulation of sNAMs in the sensory ganglia.^108^ This effect seems to be related to a decrease in the production of CCL2 in the sensory ganglia, which is a crucial chemokine in macrophages activation/infiltration.^108^ Although a direct TLR2 activation of sNAMs in the sensory ganglia may likely occur after peripheral nerve injury, we could not discard an indirect activation because TLR2 seems to be also expressed on different cells (eg, SGCs) of the sensory ganglia.^108^ Furthermore, there is evidence that TLR2 deficiency also reduced macrophages' infiltration at the nerve injury site,^184^ which could also indirectly affect neuroinflammation in the DRGs.

Another pattern recognition receptor (PRR) which has been described as important for neuropathic pain development is TLR4.^132^ Earlier studies have shown that TLR4 deficient mice are protected from peripheral nerve injury–induced neuropathic pain.^17,206^ This effect was attributed to reducing microglia activation in the spinal cord.^26,197^ However, no one has evaluated the impact of TLR4 deficiency in sNAMs activation in the sensory ganglia in models of traumatic peripheral nerve injury. There is also evidence that TLR4 mediates chemotherapy-induced peripheral neuropathic pain (eg, paclitaxel and oxaliplatin).^122,148,230^ In paclitaxel-induced neuropathic pain, the blockage of TLR4 reduced the accumulation of macrophages in sensory ganglia.^237^

Nevertheless, this was assumed as a direct effect of paclitaxel on the activation of TLR4 expressed in sensory neurons, which in turn increased the production of the macrophages chemotactic factor, CCL2.^237^ Activation of SGCs by paclitaxel in a TLR4-dependent manner and the consequent production of proinflammatory cytokines have also been suggested as a possible mechanism involved in neuropathic pain development.^226^ Although the contribution of TLR4 in sensory ganglia sNAMs for paclitaxel-induced neuropathic pain has been not investigated, it could also be an alternative. In this context, it was recently found that DRGs sNAMs-expressing TLR4 mediates the development of oxaliplatin-induced neuropathic pain.^180^ A recent study also revealed a role for TLR9 signaling in the pathophysiology of paclitaxel-induced neuropathic pain.^137^ In fact, Luo and collaborators demonstrated that paclitaxel-induced neuropathic pain was impaired in TLR9 KO mice and by the intrathecal administration of a TLR9 selective antagonist. Pain hypersensitivity was also mimicked by intraplantar and intrathecal injection of a TLR9 agonist (ODN 1826). Notably, TLR9 was found in DRG sNAMs and seemed to involve the induction of proinflammatory factors, such as cytokines.

Together with TLRs, cytoplasmic nucleotide-binding oligomerization domain-like receptors (NLRs) are the most important receptors responsible for the recognition of PAMPs or DAMPs.^19,37^ An important example of a receptor in this family is the nucleotide-binding oligomerization domain 2 (NOD2). Some studies indicate that microglial cells express NOD2,^29,31,194^ suggesting a possible role of this receptor as an innate immune sensor in the CNS. It is well established that NOD2 and TLRs act in macrophages' activation, leading to positive pressure in the proinflammatory pathways.^72^ We recently demonstrated that after peripheral nerve injury, the NOD2 expression is upregulated in sNAMs of the sensory ganglia.^175^ Using genetic inhibition of NOD2, we showed that NOD2 signaling is involved in sensory ganglia sNAMs activation/accumulation and mediates neuropathic pain development. On stimulation, NOD2 directly recruits the receptor-interacting serine/threonine-protein kinase 2, which is important for nuclear transcription factor kappa B activation and the transcription of proinflammatory genes.^75,175^ In this context, pharmacological inhibition of receptor-interacting serine/threonine-protein kinase 2 activity with a selective inhibitor (WEHI-345) also reduced the development of neuropathic pain.^175^ Altogether these studies provide consistent evidence that the manipulation of PRRs (eg, TLRs and NLRs) or their downstream signaling in sNAMs of the sensory ganglia could be explored as targets to prevent the development of peripheral neuropathic pain.

The involvement of PRRs in the activation of sensory ganglia sNAMs that account for neuropathic pain development raised the question of how these cells recognize or respond to peripheral nerve injury, which is assumed to be a sterile condition. Previous studies have suggested that damaged peripheral sensory neurons release DAMPs, such as fibronectin, high mobility group box-1, and heat shock proteins, which in turn can activate some TLRs.^201,207,208^ These DAMPs have been shown to induce further activation of numerous cell types, including glial cells and innate immune cells, which have a well-established role in the process of neuropathic pain.^65,133,178^ We recently demonstrated that neutrophil-derived S100a9, an endogenous stimulator of TLR4 signaling, plays an essential role in a model of herpetic neuralgia in a mechanism dependent on activation of TLR4 in sNAMs.^188^ Another possibility in the activation of sNAMs PRRs after nerve injury would be by PAMPs derived from microbiota. In fact, a broader role for the microbiota as a significant modulator of systemic immunity has been proposed.^99,156,173^ Microbial products derived from the microbiota can be excreted or translocated across the gut mucosa into the systemic circulation during infection or inflammation.^35,115^ These processes are involved in the development of several diseases, such as autoimmune diseases, Parkinson's disease, spinal cord injury, and neuropsychiatric disorders.^103,120,140^ For instance, bacterial microbiota–derived peptidoglycan and methylene diphosphonate are presented in rheumatoid arthritis patients' synovial tissue, contributing to the pathogenesis through NOD2 signaling activation.^90,143^ In addition, peptidoglycan-containing immune cells were detected in the CNS of multiple sclerosis patients or animals but not in healthy controls.^214,215^ Our group has shown that germ-free mice are resistant to inflammatory pain^4^. We also found that peripheral nerve injury can promote a systemic increase of an undetermined stimulant of NOD2 signaling.^188^ Thus, it is possible that after peripheral nerve injury gut microbiota–derived PAMPs (TLRs and NOD2 ligand; eg, lipopolysaccharides, peptidoglycan, and/or methylene diphosphonate) may translocate from the luminal side of the gut into the blood to distal sites (eg, sensory ganglia), activates PRRs signaling in sNAMs, and consequently contribute to the development of neuropathic pain. This hypothesis is supported by our unpublished data in which we found that there is impairment in the intestinal barrier permeability after spared nerve injury in mice. Furthermore, in a model of chemotherapy-induced neuropathic pain, there is an increase in the concentration of microbiota-derived lipopolysaccharides in the DRGs, which triggers a TLR4 dependent activation of sNAMs.^180^ Nevertheless, further studies would be important to identify the exact origin of PAMPs or DAMPs that mediate sNAMs activation in the sensory ganglia and contribute to neuropathic pain development.

### 4.2. Additional mechanisms of sensory neuron–associated macrophages activation/accumulation after peripheral nerve injury

Besides the role of PRRs in the activation/accumulation of sNAMs in the sensory ganglia after peripheral nerve injury, emerging studies were designed to find additional mechanisms explaining how distal damage to primary sensory neurons could activate sensory neurons sNAMs and consequently to the development and maintenance of neuropathic pain. Among these possible mechanisms, the most characterized are those dependent on chemokines (CCL2/CCR2 and CX3CL1/CX3CR1 pathways), cytokines (CSF1/CSFR1 axis), and microRNAs.

### 4.3. Chemokines/cytokines trigger sensory neuron–associated macrophages activation

Among the central communication systems of sNAMs and their microenvironments are the chemokine/chemokine receptors interaction. Chemokines are a vast group of peptides that act primarily to attract leukocytes to a given environment after infection or tissue damage.^169–171^ These molecules act on receptors coupled to G proteins found in different populations of circulating and resident cells. Two important chemokine axis seem to regulate sNAMs activities: (1) the CX3CL1, also known as Fractalkine, and its receptor CX3CR1^32–34^; (2) CCL2, also known as MCP-1, and its receptor CCR2 CX3CR1 is a classical marker of resident macrophages, including sNAMs, especially those originated from earlier precursors in the YS.^84,112,113^ CX3CR1-expressing sNAMs are in close contact with the cell body of sensory neurons in the sensory ganglia, which constitutively express the membrane-bound CX3CL1.^213^ The stimulation of the CX3CL1/CX3CR1 pathway in the dorsal horn of the spinal cord is a well-known mechanism involved in peripheral nerve injury–induced microglial activation/proliferation and neuropathic pain development.^34–36,213,237^ Despite all the studies that indicated that the CX3CL1/CX3CR1 pathway in microglia plays a crucial role in neuropathic pain development,^124^ none of these studies ruled out the possible role of this signaling in CX3CR1-expressing sNAMs of the sensory ganglia. In this context, after sciatic nerve injury or chemotherapy drug treatment, positive regulation of the CX3CL1/CX3CR1 axis in the sensory ganglia occurs.^28,30,213,237^ Furthermore, after peripheral nerve injury, membrane-bound CX3CL1 is reduced in sensory neurons' cell bodies, suggesting its release and action.^100,101^ In fact, neutralization of CX3CL1 in the sensory ganglia reduced chemotherapy-induced neuropathic pain^81,218^, which was associated with a reduction in the accumulation of sNAMs in the DRGs.^81^ In addition, in vincristine-induced pain, another model of CIPN, macrophages, also accumulate in the sciatic nerve and promote pain hypersensitivity in a CX3CR1-dependent manner.^161^ Therefore, the development of specific tools or approaches to investigate the particular contribution of the CX3CL1/CX3CR1 pathway in the spinal cord microglia or sNAMs in the periphery (eg, sensory ganglia or sciatic nerve) for the development of neuropathic pain are necessary.

The well-characterized chemokine that brings blood monocytes into inflamed tissues is CCL2.^116,204^ This chemokine recruits monocytes/macrophages by activating its highly affinity CCR2 receptor.^67,185^ This axis seems to play an essential role in the neuroinflammation process, including those associated with neuropathic pain development.^1,237^ In fact, mice lacking CCL2 or CCR2 are resistant to the development of neuropathic pain caused by peripheral nerve injury. Furthermore, pharmacological inhibition of CCL2 and CCR2 with neutralizing antibody or antagonist, respectively, also attenuates mechanical allodynia induced by peripheral nerve injury.^53,227^ Neutralization of the CCL2/CCR2 axis also protected from chemotherapy-induced neuropathic pain.^3,83^ These studies strongly support the role of the CCL2/CCR2 axis in the development of some types of neuropathic pain. However, the mechanisms by which the CCL2/CCR2 axis mediates neuropathic pain development are not totally clear, but they might be multiples.^227^ For instance, genetic or pharmacological inhibition of the CCL2/CCR2 pathway reduced monocytes accumulation in the sciatic nerve after traumatic nerve injury,^25,127,154,186^ suggesting a peripheral effect. On the other hand, recent data did not show any change in the accumulation of sNAMs in the sensory ganglia after peripheral nerve injury,^235^ indicating that the CCL2/CCR2 axis participates in the development of neuropathic pain would be preferentially at the local of the nerve injury. Supporting this hypothesis, perineural injection of CCL2 promotes pain hypersensitivity dependent on monocytes' recruitment.^40^ Some studies suggest a possible role for the CCL2/CCR2 pathway in the spinal cord in the pathophysiology of neuropathic pain.^92,198^ For instance, they demonstrated an increase in the expression of CCL2 by injured sensory neurons, which might be transported and released into the spinal cord, promoting the activation of CCR2-expressing glial cells.^209^ However, it is striking that in CCR2-RFP mouse, a mouse strain in which CCR2-expressing cells also express red fluorescent protein, no significant detection of CCR2+ cells was observed in the spinal cord either in naive condition or after peripheral nerve injury.^68,71^ There is also evidence suggesting CCL2 directly enhances primary sensory neurons excitability.^16,92,223^ One significant problem to address the exact role of the CCL2/CCR2 axis in the development of neuropathic pain is the lack of specific tools, especially specific antibodies, to stain CCL2 and CCR2.

Furthermore, Ccr2 null mice have a defect to mobilize monocytes from the bone marrow; thus, even in naive conditions, these animals already have fewer monocytes in the bloodstream.^232^ Noteworthy, double-blind clinical trials failed to demonstrate the efficacy of a selective and safe CCR2 antagonist in diabetes and posttraumatic neuropathic pain.^97,98^ Therefore, further preclinical studies and clinical trials are necessary to further confirm the importance of the CCL2/CCR2 axis for neuropathic pain development and also the possible mechanisms underlying.^198,199^

Besides chemokines, cytokines' role in the activation/accumulation of sNAMs in the sensory ganglia after peripheral nerve injury was recently analyzed.^235^ The specific knockdown of CSF1 in sensory neurons reduced macrophages activation/accumulation in DRGs after peripheral nerve injury and neuropathic pain.^235^ These results indicated that the production/release of CSF1 by injured sensory neurons plays a crucial role in the direct sNAMs interaction that accounts for neuropathic pain development.

### 4.4. MicroRNAs and sensory neuron–associated macrophages

Studies report that after peripheral nerve injury, there is a robust dysregulation in the expression of noncoding RNAs, including microRNAs in sensory neurons.^12,114,153,174,189^ For instance, it was found that activated or injured primary sensory neurons can release miR-21-5p as cargo in extracellular vesicles (eg, exosomes).^188^ These sensory neuron–derived exosomes and their miR-21-5p cargo mediate sNAMs activation/accumulation in DRGs. In fact, after release, miR-21-5p in extracellular vesicles is readily captured by sNAMs in the DRG that, in turn, induces the polarization of sNAMs into a pronociceptive and proinflammatory phenotype.

Aside from these studies that provide several possible mechanisms involved in activation/accumulation of sNAMs in the sensory ganglia after peripheral nerve injury and, consequently, in developing neuropathic pain, further studies will be necessary to understand these crosstalks completely. For example, studies that conditionally knockdown molecular pathways or receptors in sNAMs would be required to further understand these interaction mechanisms.

### 4.5. Sensory neuron–associated macrophages effector mechanisms mediating neuropathic pain development

Based on the evidence we have described above, it is becoming clear that peripheral macrophages (eg, sNAMs of the sensory ganglia) participate in the pathophysiological process involved in the genesis of neuropathic pain of different subtypes. Of interest to the community is how peripheral macrophages reciprocally influence sensory neurons excitability after nerve injury that may contribute to these pathological states. Two main effector mechanisms have been attributed to peripheral macrophages at the local of nerve injury and sensory ganglia in the induction of neuropathic pain: (1) production of proinflammatory/nociceptive cytokines and (2) production of reactive oxygen species that in turn trigger TRPA1 stimulation.^6,15,27,149,211,234^

Resident sNAMs together with Schwann cells are the main source of the initial cytokines/chemokines cascade responsible for the recruitment of additional leukocytes, such as neutrophils, monocytes, and lymphocytes that infiltrate the local of nerve injury.^11,25,105,127^ Besides promoting leukocytes recruitment, which amplify the inflammatory/immune process in the local nerve injury, these cytokines/chemokines may also directly enhance the excitability of primary sensory neurons.^159,191,217,218,223^ Among cytokines produced/released by macrophages in the local of nerve injury that may affect directly and/or indirectly the excitability of primary nociceptive neurons, tumor necrosis factor (TNF), IL-1β, and IL-6 are well characterized.^53,56,125,159,177,191,217,223^ Notably, the expression of proinflammatory cytokines in injured human nerve biopsies has been reported, and this response correlates with the degree of neuropathic pain.^129^

The activation phenotype of sNAMs in the sensory ganglia after peripheral nerve injury has been also associated with the production of proinflammatory cytokines.^235^ We have shown that after spared nerve injury, the activation of NOD2 signaling in sNAMs mediates neuropathic pain development in a mechanism dependent on the production of TNF and IL-1β.^175^ More recently, the CSF1/CSF1R signaling-dependent activation of sNAMs also triggers neuropathic pain through the production of IL1b.^235^ Finally, it was suggested that sNAMs-derived IL-1β stimulates brain-derived neurotrophic factor by primary sensory neurons as a possible mechanism involved in the development of neuropathic pain.^235^ Nevertheless, it is striking that sensory neurons specific knockdown of brain-derived neurotrophic factor did not affect neuropathic pain development.^42^ Thus, the role of sNAMs-derived IL-1β in the sensory ganglia for the development of neuropathic pain is still under debate. In this context, several studies have indicated that primary sensory neurons may express receptors for proinflammatory cytokines/chemokines, including for those peripheral macrophage–derived cytokines (eg, IL-1β, TNF, and IL-6 receptors).^131,138^ Based on that, several studies have analysed the possible effects of these cytokines on the excitability of primary sensory neurons.^151,159^ For example, both TNF and IL-1β are able to enhance the excitability of cultured primary sensory neurons in vitro. Nevertheless these results would be analysed with caution because normally cultures of primary sensory neurons also contain other cell subtypes such as SGCs, and these cells may also express receptors for these cytokines,^187^ hindering the interpretation of the data. One possibility to confirm the specific role of cytokines/cytokines receptor signaling directly on sensory neurons is the development of conditional animals that lack the expression of these cytokines receptors only in pain fibers. In this context, the specific knockout of gp130, a subunit of IL-6 receptor in primary nociceptive neurons, did not affect the development of neuropathic pain, suggesting no role for a direct action of IL-6 on sensory neurons in neuropathic pain^8^. Furthermore, the deletion of Il1r1 exclusively in the population of TRPV1+ nociceptors prevented the development of pathological pain in models of arthritis and multiple sclerosis.^138^ The future use of these Il1r1 conditional mice and the generation of TNF receptors conditional knockout mice in primary nociceptive neurons would be necessary to explore and confirm this possibility in models of neuropathic pain after peripheral nerve injury.

Another possible effector mechanism by which peripheral macrophages and sNAMs contribute for neuropathic pain development is through the production of ROS. For example, ROS produced by recruited monocytes into the peripheral injured nerves mediates neuropathic pain development.^40,203^ In fact, the depletion of these cells by clodronate treatment was able to attenuate the levels of hydrogen peroxide in the injured tissue, as well as nociceptive behavior.^40,95^ It also showed that monocyte-derived ROS signals through TRPA1 receptors triggering peripheral sensibilization.^7,23,40,203^ Whereas monocytes recruitment to the site of nerve injury that increase ROS production is dependent on CCL2/CCR2 signaling, there is evidence that macrophages/monocytes activation is dependent on ATR2 signaling^40,182,183^ The sciatic nerve accumulated macrophages/monocytes also promote ROS production in CX3CR1-dependent manner and mediates vincristine-induced neuropathic pain.^161^ There are several intracellular process and pathways that generate ROS, including mitochondria, xanthine oxidase, cytochrome P450 complexes, lipoxygenases, uncoupled endothelial nitric oxide synthase, and nicotinamide adenine dinucleotide phosphate oxidases. Nox-derived ROS has been implicated in the pathophysiology of neuropathic pain.^94,107^ Notably, sNAMs of the sensory ganglia express Nox2 and increase the production of ROS after peripheral nerve injury.^96^ Altogether these studies indicate that peripheral macrophage–derived ROS, including Nox2 dependent, might be an interesting target for neuropathic pain control. Based on this hypothesis, pioglitazone, a PPARγ agonist, reduces cisplatin-induced neuropathic pain by reducing ROS production in the sensory ganglia.^102^

### 4.6. Sensory neuron–associated macrophages and resolution of neuropathic pain

Concomitantly to the production of pronociceptive molecules by immune and glial cells across the pain pathway (local of injury; sensory ganglia, and spinal cord) after peripheral nerve injury, there is also evidence suggesting the production of anti-inflammatory/antinociceptive molecules.^3,5,14,20,22,52,85,144,145,168,229^ In this context, increasing evidence suggests that peripheral macrophages also play an important role in the resolution of chronic pain.^25,39^ The identification of these regulatory mechanisms in peripheral macrophages that counteract neuropathic pain would also reveal novel targets for its treatment. For instance, we recently found that at the level of sensory ganglia there is an increase in the production of IL-27 which plays a regulatory role in the development of neuropathic pain.^52^ We also showed that Il-27 counteracts neuropathic pain by acting on its receptor expressed by sNAMs that in turn stimulate the production of the antinociceptive cytokine IL-10.^52^

Endogenous cannabinoids produced in the periphery and the CNS are important components of endogenous analgesia.^5,109,150,168,172,212^ For example, mice deficient in CB2 receptor showed enhanced pain hypersensitivity in models of neuropathic pain.^167^ The mechanisms underlying the exacerbation of neuropathic pain in CB2 receptor null mice was recently investigated.^150^ Notably, specific deletion of CB2 receptors in myeloid cells, especially in peripheral monocytes and sNAMs of the sensory ganglia, but not in neurons, also enhance neuropathic pain to the same level of whole-body deletion.^150^ These results indicate that CB2 receptor signaling in peripheral macrophages limits the development of peripheral nerve injury–induced neuropathic pain. The mechanisms by which CB2R signaling modulates peripheral macrophages is not totally clear but seems to involve an increase in leptin signaling.^150,157^ It could be also due to a reduction in the production of other pronociceptive mediators derived from peripheral macrophages. In fact, activation of CB2 receptors in macrophages reduced the production of proinflammatory cytokines (TNF and IL-1β) and ROS.^73,135^ Thus, the development of CB2R agonists acting specifically in the periphery would be an interesting approach to target macrophages and to inhibit neuropathic pain development.

## 5. Conclusion remarks

**Figure 1.**
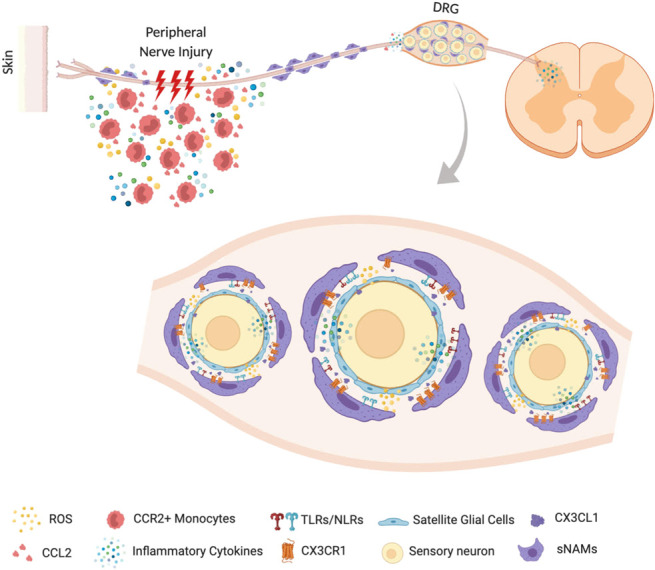
Representative illustration of the role of peripheral macrophages in the development of neuropathic pain. In the injured peripheral nerves, resident cells (Schwann cells, sNAMs) produced proinflammatory mediators, such as cytokines/chemokines which mediate the recruitment of additional leukocytes (eg, blood CCR2+ monocytes) and then more pronociceptive mediators are produced. This soup of proinflammatory cytokines amplifies the sensitization of primary sensory neurons and accounts for neuropathic pain development. In addition, after peripheral nerve injury, there is also accumulation/activation of sNAMs in the sensory ganglia. These cells also mediate the development of neuropathic pain through the production of cytokines (eg, IL-1β) and ROS. The possible molecular mechanisms involved in the activation of sNAMs in the sensory ganglia are also depicted. sNAMs, sensory neuron–associated macrophages.

In summary, this review pointed out the crucial participation of peripheral macrophages, especially sNAMs located in the sensory ganglia, for the development of neuropathic pain. It also described the cellular and molecular mechanisms involved in peripheral macrophages (eg, sensory ganglia sNAMs) activation/accumulation and effector functions after peripheral nerve injury that account for neuropathic pain development (Fig. 1). In conclusion, these mechanisms could be explored as possible targets for the development of novel drugs to treat neuropathic pain.

## Disclosures

The authors have no conflicts of interest to declare.

T.M. Cunha receives funding from the São Paulo Research Foundation (FAPESP) under grant agreements 2013/08216-2 (Center for Research in Inflammatory Disease) and 2017/50419-9. C.E.A. Silva and R.M. Guimarães have a PhD fellowship from FAPESP (2020/05446-0 and 2019/13829-0).
